# *In vitro* antioxidant capacity of *Corryocactus brevistylus* (sanky) and its effect on the pancreas morphology of alloxan-induced diabetic rats

**DOI:** 10.17843/rpmesp.2023.403.12481

**Published:** 2023-09-28

**Authors:** Liz Delia Arostegui-Faustino, Oscar Gustavo Huamán-Gutiérrez

**Affiliations:** 1 Hospital de la Solidaridad-Los Olivos, Lima, Peru. Hospital de la Solidaridad-Los Olivos Lima Peru; 2 Department of Nutrition, School of Medicine, Universidad Nacional Mayor de San Marcos, Lima, Peru. Universidad Nacional Mayor de San Marcos Department of Nutrition School of Medicine Universidad Nacional Mayor de San Marcos Lima Peru

**Keywords:** Antioxidant Capacity, Hypoglycemic, Pancreas, Medicinal Plants

## Abstract

**Objective.:**

To determine the in vitro antioxidant capacity of *Corryocactus brevistylus* and its effect on glycemia and the pancreas of alloxan-induced diabetic rats.

**Materials and methods.:**

The antioxidant capacity of the hydroethanolic extract of sanky (HEES) was evaluated by assessing its ability to reduce 2,2-diphenyl-1-picrylhydrazyl (DPPH) and ferric ion (FRAP). We used thirty adult rats, which were induced to diabetes with two doses of alloxan (80mg/kg). Rats were distributed into 5 groups (n=6), all groups received treatment by orogastric route for eight days. Group I received water, group II received metformin 14mg/kg and groups III, IV and V received sanky juice at 1.0; 4.0 and 16 mL/kg, respectively. Glycemia was evaluated by the rapid method (glucometer) (first and eighth day). After treatment, the animals were sacrificed and the pancreas was removed for histopathological study.

**Results.:**

The antioxidant capacity of HEES by DPPH showed an IC_50_ of 0.77 mg/mL; the FRAP method showed a TEAC-FRAP of 22.31µg/mg. Glycemia decreased on the eighth day of treatment, with respect to the first day; a decrease in glycemia was also found in groups III-V, when compared to group I. Histologically, groups I-II presented severe atrophy and moderate necrosis of the islets of Langerhans; groups IV-V presented hypertrophy and mild multifocal necrosis at the islet level.

**Conclusions.:**

The extract of sanky showed antioxidant capacity in vitro and the juice exerts a hypoglycemic and protective effect on the pancreas.

## INTRODUCTION

The pancreas is a single, retroperitoneal organ, located in the abdomen at the level of the first and second lumbar vertebrae and behind the stomach, measuring between 16 to 20 cm and weighing between 85-120 g ^1^. It has two important functions, the exocrine function which is fundamental for the digestion process, secreting the enzymes lipase and amylase, and the endocrine function, which is responsible for the production of hormones, mainly insulin, a fundamental hormone for the regulation of blood sugar levels ^2^.

Diabetes is a chronic metabolic disease characterized by hyperglycemia and disorders regarding the metabolism of carbohydrates, fats and proteins; pharmacological treatment is long and complex. The rates are increasing despite efforts to reduce its impact, so it is important to improve adherence to pharmacological and non-pharmacological treatment ^3^.

Worldwide, the prevalence of type 2 diabetes *mellitus* (DM2) in people over 18 years of age has increased from 4.7% in 1980 to 8.5% in 2014, noting that the lower the family income and education, the higher the risk of developing DM2 ^4^. In Peru, the prevalence of DM2 reaches 7%; however, it is 8.2%, in the coastal region, being lower in the highlands with 4.5% and jungle with 3.5%; but it should be highlighted that the prevalence reaches 8.4% in Metropolitan Lima ^5^.

The use of medicinal plants for therapeutic purposes is a practice that has been growing since ancient times. In Peru there is a great variety of plants with different attributed properties, such as anti-inflammatory, antioxidant and hypoglycemic properties, which could be related to the content of phenolic compounds such as caffeic acid and p-coumaric acid, which play a role during the digestion processes, both of carbohydrates and lipids ^6^.

Sanky is one of the foods with beneficial properties, it is a fruit from the Peruvian Andes that grows in the southern part of the country, which has been attributed to have beneficial effects, such as functional and therapeutic properties ^7^. The sanky pulp has a high content of reducing sugars, fiber and vitamin C, while the peel, in addition to the above mentioned, has phenolic acids, lactones, steroid triterpenes, minerals such as calcium, potassium, phosphorus and magnesium ^8^.

In 2022, Lopez studied the lipid-lowering effect of sanky, finding that at a dose of 10 mL/kg of sanky juice, triglyceride levels decreased and HDL levels increased ^9^. Lipe in 2016 evaluated the hepatoprotective effect of this fruit, and reported that the *Corryocactus brevistylus* (sanky) juice had a protective effect on the liver, according to biochemical indicators of NP-SG (non-protein sulfhydryl groups), in mice with ethanol-induced liver damage ^10^.

This study was designed to evaluate the use of sanky in certain diseases, however, there was no scientific evidence on its properties in humans. The results of the antioxidant capacity reflected by the DPPH and FRAP method, the hypoglycemic effect reported on the eighth day of treatment and the characteristics of pancreatic tissue preservation, provide the basis for a new alternative that could be included in the diet of people with hyperglycemia. At the same time, we highlight the importance of the benefits of natural products such as the absence of adverse effects in comparison with conventional medicine, which would contribute to generate a new, accessible and safe alternative to go along with the treatment. This would be an option for creating a “nutraceutical” product that would benefit the overall population as well as people with chronic diseases such as diabetes by providing scientific bases on the effective and safe use of sanky ^11^.

Therefore, our study aimed to evaluate the *in vitro* antioxidant capacity of the hydroalcoholic extract and the effect of its juice on glycemia and pancreas of alloxan-induced diabetic rats.

**KEY MESSAGES**

Motivation for the study. We aimed to assess the use of sanky in certain diseases; however, there was no scientific evidence about such effects in our organism.

Main findings. The antioxidant capacity of HEES by DPPH, showed an IC_50_ of 0.77 mg/mL, and the TEAC-FRAP was 22.31µg/mg by the FRAP method. Glycemia decreased on the eighth day of treatment, when compared to the first day, it also decreased in groups III-V, when compared to group I.

Implications. The sanky extract showed antioxidant capacity *in vitro* and the juice exerts a hypoglycemic and protective effect on the pancreas.

## MATERIALS AND METHODS

### Study design and setting

This is a purely experimental study, with control group as well as pre- and post-tests ^12^.

### Collection of sanky fruit

Mature sanky (*Corryocactus brevistylus*) fruits were collected from the Lucanas district (14° 37’19.6’’S and 74° 13’55.3’’W), Ayacucho, Peru. The fruits were stored in wooden boxes and completely sealed for later shipment to Lima. The fruits were identified by the Natural History Museum of the Universidad Nacional Mayor de San Marcos as *Corryocactus brevistylus* (certificate N°018-USM-MHN-2022).

### Juice preparation

To obtain the juice, ripe sanky fruits in good condition were duly cleaned, washed and peeled, then cut into halves to extract the edible part. They were placed in the extractor (Oster brand), which produced approximately 20 mL of juice per fruit; this procedure was performed early in the day, each of the days of sample administration.

### Obtention of the hydroethanolic extract

To obtain the dry sample (hydroethanolic extract), 1 kg of the edible part of the fruit was extracted, then it was chopped and macerated in 70% ethanol. This preparation was kept in an amber-colored bottle at room temperature for seven days, with manual shaking in a circular motion for 15 minutes each day. At the end of the maceration period, it was filtered with filter paper (Whatman 110 mm), due to the persistence of turbidity, the filtrate was centrifuged at 3500 rpm for five minutes. The obtained solution was evaporated to dryness in an oven at 40 °C (MMM-German ECCOEL). The dry extract was used for the determination of *in vitro* antioxidant capacity ^13^. This form of extract allows obtaining polar substances, which possess beneficial activities such as antioxidant properties.

### Determination of antioxidant activity *in vitro*

*DPPH method*

According to Brand-Williams *et al.*^14^, we prepared dilutions of the hydroethanolic extract at 0.550, 0.826 and 1.651 mg/mL (R2=0.991). Trolox (ALDRICH) was used as antioxidant standard at concentrations of 1.25; 2.5; 5; 10 µg/mL (R2=0.9998). We mixed 0.4 mL of the dilutions (extract and standard) with 0.80 mL of DPPH (ALDRICH) 2 mg% (50.7 µM) and left it to stand for 30 minutes protected from light. Then they were read in spectrophotometer (GENESYS 10s) at 517 nm. The results were expressed as mean inhibitory concentration (IC_50_) and antioxidant capacity in Trolox equivalent (TEAC-DPPH).

*FRAP method*

According to Benzie *et al*. ^15^, we prepared dilutions of the hydroethanolic extract at 1.40; 2.18 and 4.20 mg/mL (R2=0.9981). We used Trolox (ALDRICH) as the standard at concentrations of 25; 50; 75 and 100 µg/mL (R2=0.9993). Readings were performed after 10 minutes at a wavelength of 593 nm. A curve was prepared for FeSO_4_ 100 at 750 µM (R2=0.9908). The results were expressed in µmol equivalent FeSO_4_ per g of hydroethanolic extract and µmol equivalent Trolox per mg of hydroethanolic extract.

### Conditioning and acclimatization of animals

The rats underwent a seven-day acclimatization period, under controlled conditions of temperature (22 ± 3 °C) and relative humidity (between 40-60%). They were distributed in 10 cages with metal grids (50 cm long, 30 cm wide and 25 cm high); three rats were placed in each cage and were exposed to 12-hour cycles of light and darkness, with water (Cielo® brand) *ad libitum* and balanced feeding.

### Study population and sample

The population consisted of 30 male Holtzman *Rattus norvegicus* albino rats weighing 190 ± 7.4 g, acquired from the biotherium of the National Institute of Health. The rats were healthy and three months old. Two rats with aggressive behavior and signs of previous manipulation were excluded.

*Induction of experimental diabetes and description of the experiment*

In order to induce experimental diabetes, alloxan was administered intraperitoneally in two doses of 80 mg/kg dissolved in 0.3 M citrate buffer pH 4.5, with a space of 48 hours between the first and second doses. Rats whose glucose levels exceeded 200 mg/dL after a 12-hour fast were included ^16^.

For the experimental phase, the animals were distributed in five groups (n=6), receiving the following treatment for eight days, via orogastric route:

Group I: Water

Group II: metformin 14 mg/kg b.w.

Group III: fruit juice 1 mL/kg b.w.

Group IV: fruit juice 4 mL/kg b.w.

Group V: fruit juice 16 mL/kg b.w.

The blood glucose level was evaluated on the first and eighth day, after fasting for 12 hours, making an incision with a scalpel in the tip of the tail, after cleaning and disinfection, discarding the first drop of blood, Glucose measurement was performed in duplicate, in a fast determination equipment (ACCU-CHEK Instant).

Once the experimental process was finished, the animals were euthanized with sodium pentobarbital (50 mg/kg intraperitoneally) ^17^, then a laparotomy was performed to remove the pancreas.

*Histological analysis*

Pancreatic tissue was preserved in 10% buffered formalin solution (0.05 mol/L phosphate buffer at pH 7.4) for subsequent histopathological study. The samples were then paraffinized, and hematoxylin-eosin staining was used to characterize the microscopic alterations. The samples were evaluated at 40X by a medical-pathologist professional; the damage evaluation was expressed in an ordinal scale: mild (+), moderate (++) and severe (+++).

### Statistical analysis

The data were analyzed in the SPSS version 27 program. We evaluated normality with the Shapiro Wilk test with a value of p>0.05; we used parametric statistics. The mean and standard deviation were used as a measure of central tendency and dispersion. The analysis of variance test (ANOVA) and Levene’s statistic were used, and then comparisons were made using Tukey’s test.

### Ethical Aspects

Our research complied with the aspects contemplated by Law No. 30407 “Animal Protection and Welfare Law”, according to Article 19 of Chapter V “Animal ownership, protection and management”, which guarantee the greatest protection against physical pain, based on good management practices, biosafety and bioethics according to the experimented animal species ^18^. In addition, we complied with two of the three animal experimentation principles, proposed by Russell and Burch, which are Reduce and Refine. This study has the approval of the Research Ethics Committee of the Faculty of Medicine of the Universidad Nacional Mayor de San Marcos (code No. 0053-2022).

## RESULTS

### *In vitro* antioxidant capacity

**Figure 1:**
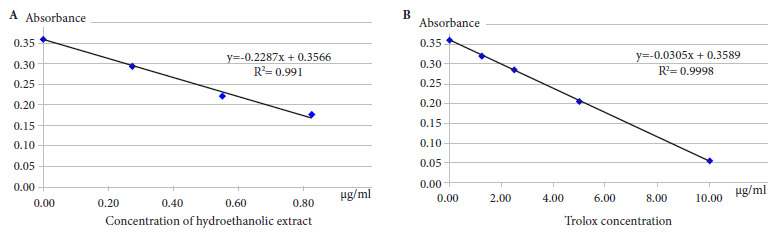
2,2-diphenyl-1-picrylhydrazyl reduction curves for (A) Hydroethanolic extract (B) Trolox.

We assessed the antioxidant capacity with the DPPH method, and it showed that the extract had an IC_50_ of 0.77 mg/mL, while the IC_50_ for Trolox was 5.86 µg/mL, giving a TEAC-DPPH of 7.61 µg/mg of extract (Figure 1).

**Figure 2:**
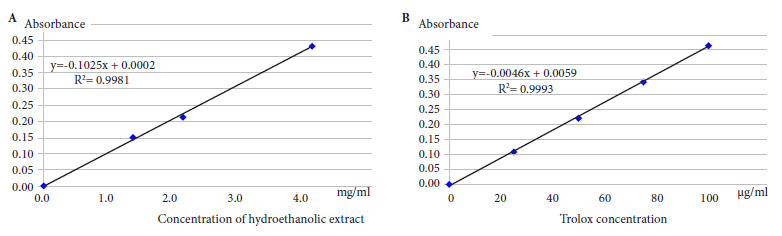
FRAP reduction curve for (A) Hydroethanolic extract (B) Trolox.

By using the FRAP method, we found that the extract at 2.93 mg/mL and Trolox at 66.5 µg/mL expressed antioxidant activity equivalent to 328.7 µM Fe^2+^, the TEAC-FRAP being 22.31 µg/mg extract (Figure 2).

### Hypoglycemic effect

Prior to the induction period with alloxan, the rats showed a fasting glucose level of 64.8 ± 10.4 mg/dL (Shapiro Willk p>0.05).

Glycemia levels decreased in groups II to V at day 8 when compared to the first day of treatment. We found that on the last day of treatment the groups that received metformin and juice at different doses showed a lower glucose level when compared to group I, with groups IV and V showing lower levels (Table 1).

**Table 1:** Glucose levels according to treatment group in rats with alloxan-induced diabetes.

Groups	Treatment	Glucose (mg/dL)	
		Day 1*	Day 8**
		Mean ± SD	Mean ± SD
Group I	Water 2 mL	496 ± 92.1	452 ± 67.8
Group II	Metformin 14 mg/kg	487 ± 81.0	139 ± 28.3^(a)(c)^
Group III	Sanky juice 1 mL/kg	431 ± 118	286 ±39.7^(a)(d)^
Group IV	Sanky juice 4 mL/kg	327 ± 75.2	114 ±13.0^(a)(b)(c)^
Group V	Sanky juice 16 mL/kg	393 ± 126	104 ±5.73^(a)(b)(c)^

SD: standard deviation.

*ANOVA test=p>0.05, ** ANOVA test=p<0.05.

Tukey’s test and ANOVA:

* ^a)^ p<0.01; compared to group I.

* ^b)^ p<0.01; compared with group III.

* ^c)^ p<0.01; compared to day 1

* ^d)^ p<0.05; compared to day 1

### Results of the histopathological study

The findings in the pancreatic tissue of experimental rats with alloxan-induced diabetes at the histological level are as follows: 

*Group I*

**Figure 3:**
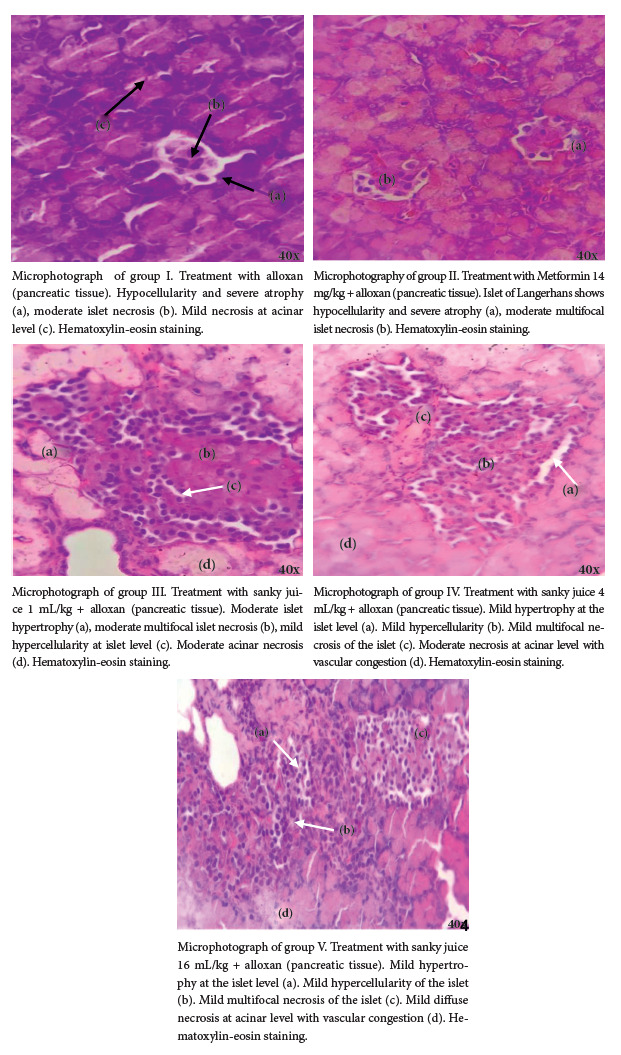
Microphotograph of pancreatic tissue in the treatment groups.

Hypocellularity and severe atrophy were found at the level of the islets of Langerhans (+++), moderate multifocal islet necrosis (++) was observed as well. We also found mild necrosis (+) at the acinar level, along with vascular congestion (Figure 3).

*Group II*

Hypocellularity and severe atrophy were observed at the level of the Islets of Langerhans (+++), as well as moderate multifocal necrosis of the islet of Langerhans (++). We also found that the pancreatic acini had moderate diffuse necrosis (++) (Figure 3).

*Group III*

Moderate hypertrophy was found at the islet level (++) and unlike the previous groups, group III showed mild hypercellularity at the level of the islets of Langerhans (+), as well as moderate multifocal necrosis of the islet (++), and at the acinar level, we observed moderate diffuse necrosis (++) accompanied by vascular congestion (Figure 3).

Group IV

This group was characterized by the presence of hypertrophy and mild hypercellularity at the level of the islets of Langerhans (+), mild necrosis (+) at the multifocal level, as well as moderate diffuse necrosis (++) and vascular congestion at the acinar level (Figure 3).

Group V

In this group, the histological section showed mild hypercellularity (+) and mild hypertrophy in the islet of Langerhans (+), mild necrosis at multifocal level (+), as well as mild necrosis (+) and vascular congestion at acinar level (Figure 3).

## DISCUSSION

Our study showed the antioxidant capacity of the extract of sanky pulp (*Corryocactus brevistylus*) by the DPPH method; the extract had an IC_50_ of 0.77 mg/mL, and the IC_50_ for trolox was 5.86 µg/mL, resulting in a TEAC-DPPH of 7.61 µg/mg of extract. By applying the FRAP method, the extract at 2.93 mg/mL presented an antioxidant activity equivalent to 328.7 µM Fe^2+^, the TEAC-FRAP being 22.31 µg/mg extract. The hypoglycemic effect of *Corryocactus brevistylus* juice was also assessed. A hypoglycemic effect was found at doses of 4 and 16 mL/kg b.w. (group IV and V, respectively) in male Holtzman strain albino rats with experimental diabetes mellitus, when compared to the first day of treatment in a period of eight consecutive days. As time passed by, the hypoglycemic effect of the sanky increased in both groups, on the other hand the dose of 1 mL/kg b.w. of group III, was not sufficient to normalize the levels of glycemia. Similarly, hypertrophy and mild necrosis were found in the islets of Langerhans in the rats of groups IV and V compared to the control group that did not receive Sanky treatment; hypocellularity and severe atrophy were found at the level of the islets of Langerhans.

This protective effect found in the experimental groups who received sanky could be due to the great *in vitro* antioxidant capacity of the dry extract. The DDPH method showed an IC_50_ of 0.77 mg/mL, which is equivalent to TEAC-DPPH of 7.61 µg/mg of extract, this result could be related to the presence of metabolites present in the fruit, as detailed by Obregón *et al*., they found that the sanky had 0.57 vitamin C mg/g of fruit, in addition to the presence of polyphenols; these compounds have been studied for their ability to neutralize free radicals ^8^. Similarly, Matos *et al*., using the same method (DPPH), found that the higher the phenol content, the higher the antioxidant capacity, reporting that antioxidant activity depends on the concentration of phenolic compounds in the extract ^19^.

Nolazco and Guevara found that sanky pulp had a Trolox equivalence of 474.8 µg/g of sample, showing good antioxidant capacity, due to its vitamin C content found (57.1 mg/100 g of sample), the authors stated that sanky has higher ascorbic acid content compared to other citrus fruits of higher consumption ^20^.

We evaluated the effect of sanky juice on the glycemia of rats induced with alloxan and the effect it causes on the morphology of the pancreas. We found that the glycemia levels after administering alloxan intraperitoneally 80 mg/kg b.w. were in accordance with the proposed methodology, achieving a glucose greater than 200 mg/dL. It was also observed that group I (control), maintained high values from the beginning of the induction until the end of the treatment, these values are related to the effects of alloxan, since it is a diabetogenic agent widely used to produce experimental diabetes in animals, due to its action mechanism on the β cells of the pancreas, increasing the levels of glucose in plasma ^21^.

The results obtained by group I (control) are related to those described by Vilches *et al*., who found that the administration of alloxan at a single dose of 100 mg/kg induced hyperglycemia with values above 200 mg/dL, maintaining these values during the seven days of treatment ^22^. Sosa *et al*. carried out the induction at a dose of 150 mg/kg of alloxan to achieve experimental diabetes with glucose higher than 250 mg/dL, finding significant differences at 30 min after the ingestion in the control group and the groups with treatments, but the variation of glucose decreased as time went by, so that there were no significant differences in the percentage of variation between the doses at 120 min postprandial, which indicates that the experimental animals had returned to their basal state ^23^.

A study on diabetic rats induced with alloxan and treated with insulin reported severe atrophy at the level of pancreatic islets, additionally, this same group showed a significant increase in the number of pancreatic cells (hypercellularity) ^24^, which differs from our research, se found a decrease of pancreatic cells (hypocellularity) in the control group and in the group with metformin treatment. Our results show, at histological level, severe atrophy in group I at the level of the Islets of Langerhans. The damage described in group I was also observed in the group of rats that received metformin, a drug used as a hypoglycemic agent, which also presented severe atrophy at the level of the islets.

Rats treated with sanky juice at high doses of 4 mL/kg and 16 mL/kg showed increased protection of pancreatic cells, and mild hypertrophy at the islet level, demonstrating that this product, in addition to lowering blood glucose levels, would provide better protection to pancreatic cells.

The effect of sanky juice on pancreatic cells could be due to its antioxidant effect, as well as its metabolite content, giving it great nutritional value as a possible functional food to attenuate the deleterious effects of free radical generation through its antioxidant capacity, as reported by Balvin in 2021 ^25^.

The group that received, in addition to alloxan, the sanky at a minimum dose of 1 mL/kg, showed signs of moderate necrosis, which would indicate minimal protection as opposed to the rats that received the highest dose, which showed mild atrophy in the pancreatic cells.

Our results cannot be extrapolated to humans because this study was carried out on experimental animals. Despite the reported potential benefits of sanky, further research is needed. It is also important to identify the bioactive compounds present in the sanky, as well as the antioxidant effect it presents; therefore, it is necessary to determine the metabolite responsible for the beneficial effects. It is worth mentioning that antioxidants are present in many foods, and according to the literature, antioxidants can neutralize the excess of free radicals during the oxidative activity of the organism, acting as the first line of defense in living beings; however, an imbalance between endogenous antioxidants and free radicals (oxidative stress) is associated with several diseases ^26^.

In view of the above, we can highlight the sanky is a fruit with high beneficial properties for the body, being the basis for the development of derivative products that would benefit the population in general, as well as nutraceutical derivatives, creams or supplements that retain their antioxidant properties, and that is easily accessible to the population.

We can conclude that sanky has an antioxidant effect *in vitro*, as well as a hypoglycemic and protective effect on pancreatic tissue at doses of 4 and 16 mL/kg at the pancreatic level in rats induced to diabetes with alloxan.
