# Supplementary material for: In vitro antioxidant capacity of Corryocactus brevistylus (sanky) and its effect on the pancreas morphology of alloxan-induced diabetic rats
Source: Rev Peru Med Exp Salud Publica. 2023 Sep 28;40(3):317–24. doi: 10.17843/rpmesp.2023.403.12481 (PMC10953654; doi:10.17843/rpmesp.2023.403.12481)
Supplement: Translation

# Translation: Capacidad antioxidante *in vitro* del *Corryocactus brevistylus* (sanky) y su efecto en la morfología del páncreas de ratas diabéticas inducidas con aloxano

**Authors:** Liz Delia Arostegui-Faustino, Oscar Gustavo Huamán-Gutiérrez

**Affiliations:** 1 Hospital de la Solidaridad-Los Olivos, Lima, Perú.; 2 Departamento de Nutrición, Facultad de Medicina, Universidad Nacional Mayor de San Marcos, Lima, Perú.

**Keywords:** Capacidad Antioxidante, Hipoglicemiante, Páncreas, Plantas Medicinales

## Abstract

**Objetivo.:**

Determinar la capacidad antioxidante *in vitro* del *Corryocactus brevistylus* y su efecto sobre la glicemia y páncreas de ratas diabéticas inducidas con aloxano.

**Materiales y métodos.:**

Se evaluó la capacidad antioxidante del extracto hidroetanólico de sanky (EHES) mediante la capacidad de reducir el 2,2-difenil-1-picrilhidracilo (DPPH) y la capacidad de reducir el ion férrico (FRAP). Se utilizaron 30 ratas adultas inducidas a diabetes con dos dosis de aloxano (80mg/kg), formándose cinco grupos (n=6), recibiendo los tratamientos vía orogástrica durante ocho días, el grupo I (agua), II (metformina 14mg/kg), grupos III-IV-V zumo de sanky a 1,0; 4,0 y 16 mL/kg, respectivamente. La glicemia fue evaluada por el método rápido (glucómetro) (primer y octavo día). Terminado el tratamiento los animales fueron sacrificados y se les extrajo el páncreas, para su estudio histopatológico.

**Resultados.:**

La capacidad antioxidante del EHES mediante el DPPH, mostró un IC_50_ de 0,77 mg/mL, y por el método FRAP se observó el TEAC-FRAP de 22,31µg/mg. La glicemia disminuyó en el octavo día de tratamiento, respecto al primer día; también se observó disminución de la glicemia en los grupos III-V, respecto al grupo I. A nivel histológico los grupos I-II presentaron atrofia severa y necrosis moderada de los islotes de Langerhans; los grupos IV-V presentaron hipertrofia y necrosis leve multifocal a nivel del islote.

**Conclusiones.:**

El extracto de sanky presenta capacidad antioxidante *in vitro* y el zumo ejerce un efecto hipoglicemiante y protector en páncreas.

## INTRODUCCIÓN

El páncreas es un órgano único, retroperitoneal, localizado en el abdomen a nivel de la primera y segunda vértebra lumbar y por detrás del estómago, mide entre 16 a 20 cm y pesa entre 85-120 g ^1^. Posee dos funciones importantes, la función exocrina que es fundamental en el proceso de la digestión, secretando las enzimas lipasa y amilasa, mientras que la función endocrina se encarga de la producción de hormonas, principalmente la insulina, que es fundamental para la regulación de los niveles de azúcar en la sangre ^2^.

La diabetes es una enfermedad crónica de tipo metabólico caracterizada por hiperglucemia con disturbios en el metabolismo de los carbohidratos, grasas y proteínas, el tratamiento farmacológico es largo y complejo. A pesar de los esfuerzos para disminuir el impacto, los índices se van incrementando, por ello es importante mejorar la adherencia al manejo farmacológico y no farmacológico ^3^.

En el mundo, la prevalencia de diabetes *mellitus* tipo 2 (DM2) en personas mayores de 18 años, ha aumentado de 4,7% en 1980 a 8,5% en 2014, observándose que, a menor ingreso familiar y educación, el riesgo de desarrollar DM2 es mayor ^4^. En el Perú, la prevalencia de DM2 alcanza el 7%; sin embargo, la prevalencia en la región costa es de 8,2%, siendo menor en la sierra con un 4,5% y selva con un 3,5%; pero hay que resaltar en Lima Metropolitana la prevalencia alcanza un 8,4% ^5^.

El uso de plantas medicinales con fines terapéuticos es una práctica que se ha venido incrementado desde la antigüedad, en el Perú existe una gran variedad de plantas, a las cuales se les atribuyen distintas propiedades, como antiinflamatorio, antioxidante e hipoglicemiante, dichas propiedades podrían estar relacionadas con el contenido de compuestos fenólicos presentes como el ácido cafeico y el ácido p-cumárico, que actúan en los procesos de digestión, tanto de carbohidratos como de lípidos ^6^.

Entre los alimentos que tienen propiedades benéficas está el sanky, este es un fruto de los Andes peruanos, que crece en la zona sur del país, al cual se le atribuyen efectos benéficos, como propiedades funcionales y terapéuticas ^7^. La pulpa de sanky presenta alto contenido de azúcares reductores, fibra y de vitamina C, mientras que la cáscara, además de lo mencionado, presenta ácidos fenólicos, lactonas, triterpenos esteroides, minerales como calcio, potasio, fósforo y magnesio ^8^.

En un estudio realizado por López en el 2022, evaluó el efecto hipolipemiante del sanky, encontrando que a dosis de 10 mL/kg de zumo de sanky, los niveles de triglicéridos fueron disminuyendo, y los niveles de HDL se vieron incrementados ^9^. Lipe en el 2016, evaluó el efecto hepatoprotector de esta fruta, y observó que la administración del zumo de *Corryocactus brevistylus* (sanky) ejerció un efecto protector en el hígado, según los indicadores bioquímicos de GS-NP (grupos sulfhidrílos no proteicos), en ratones con daño hepático inducido con etanol ^10^.

El presente estudio surgió de la inquietud por evaluar una práctica que se venía dando sobre el uso del sanky en ciertas enfermedades, sin embargo, no había evidencias científicas sobre sus propiedades en nuestro organismo. Con los resultados de la capacidad antioxidante reflejado por el método DPPH y FRAP, el efecto hipoglicemiante observado al octavo día de tratamiento y las características de conservación del tejido pancreático, se estaría sentando las bases de una nueva alternativa que podría ser incluido en la alimentación de aquellas personas que presenten hiperglicemia. A su vez se estaría resaltando la importancia de los beneficios que presentan los productos naturales como la ausencia de efectos adversos en comparación con la medicina convencional, por lo que contribuirían a generar una nueva alternativa que acompañe al tratamiento, siendo accesible y seguro. Esta seria una opción para la elaboración de un producto «nutracéutico» que beneficiaría a la población en general y directamente a quienes padecen de diversas enfermedades crónicas como la diabetes al aportar bases científicas sobre el uso eficaz y seguro del sanky ^11^.

Por lo expuesto anteriormente el presente estudio tuvo la finalidad evaluar la capacidad antioxidante *in vitro* del extracto hidroalcohólico y el efecto del zumo sobre la glicemia y páncreas de ratas diabéticas inducidas con aloxano.

**MENSAJES CLAVE**

Motivación para realizar el estudio. Evaluar una práctica que se venía dando sobre el uso del sanky en ciertas enfermedades; sin embargo, no había evidencias científicas sobre dichos efectos en nuestro organismo.

Principales hallazgos. La capacidad antioxidante del EHES mediante el DPPH, mostró un IC_50_ de 0,77 mg/mL, y por el método FRAP se observó el TEAC-FRAP de 22,31µg/mg. La glicemia disminuyó en el octavo día de tratamiento, respecto al primer día, y también se observó disminución en los grupos III-V, respecto al grupo I.

Implicancias. El extracto de sanky presenta capacidad antioxidante *in vitro* y el zumo ejerce un efecto hipoglicemiante y protector en el páncreas.

## MATERIALES Y MÉTODOS

### Diseño y entorno del estudio

El estudio es de tipo experimental puro, con grupo control y pre y posprueba ^12^.

### Recolección del fruto del sanky

Los frutos maduros de sanky (*Corryocactus brevistylus*) fueron recolectados del distrito de Lucanas (14° 37´19,6´´S y 74° 13´55,3´´ O), región de Ayacucho en Perú. Los frutos fueron almacenaron en cajas de madera y selladas completamente para su posterior envío a Lima. El fruto fue identificado por el Museo de Historia Natural de la Universidad Nacional Mayor de San Marcos como *Corryocactus brevistylus* (certificado N°018-USM-MHN-2022).

### Preparación del zumo

Para la obtención del zumo se tomaron los frutos maduros de sanky que se encontraban en buen estado, los cuales fueron debidamente limpiados, lavados y pelados, luego se procedió a cortarlos en mitades para extraer la parte comestible, y pasarlos con mayor facilidad por el extractor (marca Oster), donde se obtuvo aproximadamente 20 mL del zumo por fruto; este procedimiento se realizó a tempranas horas del día, cada uno de los días de administración de la muestra.

### Obtención del extracto hidroetanólico

Para la obtención de la muestra seca (extracto hidroetanólico), se tomó 1 kg de la parte comestible de la fruta, luego fue picada, para posteriormente ser macerada en etanol al 70%, este preparado fue conservado en un frasco de color ámbar a temperatura ambiente durante siete días, con agitado manual, de forma circular durante 15 minutos cada día. Terminado el periodo de maceración, se filtró con papel filtro (Whatman 110 mm), debido a la persistencia de turbidez el filtrado fue centrifugado a 3500 rpm por cinco minutos. La solución obtenida fue evaporada hasta sequedad en una estufa a 40 °C (MMM-alemán ECCOEL). El extracto seco obtenido se empleó para la determinación de la capacidad antioxidante *in vitro*^13^. Esta forma de extracto permite obtener sustancias polares, de las cuales varias poseen actividades benéficas como la de antioxidante.

### Determinación de la actividad antioxidante* in vitro*

*Método DPPH*

Según Brand-Williams *et al.*^14^. Se preparó diluciones del extracto hidroetanólico 0,550; 0,826 y 1,651 mg/mL, presentando R2=0,991; y como estándar de antioxidante se utilizó Trolox (ALDRICH) a concentraciones de 1,25; 2,5; 5; 10 µg/mL; presentando un R2=0,9998. Se hizo reaccionar 0,4 mL de las diluciones (extracto y estándar) con 0,80 mL de DPPH (ALDRICH) 2 mg% (50,7 µM), dejando reposar por 30 minutos protegido de la luz. Luego fueron leídas en espectrofotómetro (GENESYS 10s) a 517 nm. Los resultados fueron expresados en concentración inhibitoria media (IC_50_) y en capacidad antioxidante en equivalente de Trolox (TEAC-DPPH).

*Método FRAP*

Según Benzie *et al*. ^15^. Se preparó diluciones del extracto hidroetanólico a 1,40; 2,18 y 4,20 mg/mL, presentando un R2=0,9981; y como estándar se utilizó Trolox (ALDRICH) a concentraciones de 25; 50; 75 y 100 µg/mL (R2=0,9993); las lecturas fueron realizadas después de los 10 minutos a una longitud de onda de 593 nm. Se preparó una curva de FeSO4 100 a 750 µM, presentando un R^2^=0,9908. Los resultados fueron expresados en µmol equivalente FeSO_4_ por g de extracto hidroetanólico y µmol equivalente Trolox por mg de extracto hidroetanólico.

### Condicionamiento y aclimatación de animales

Las ratas se mantuvieron un periodo de aclimatación de siete días, en condiciones controladas de temperatura (22 ± 3 °C) y humedad relativa (entre 40-60%), se distribuyeron en 10 jaulas provistas de rejillas metálicas de 50 cm de largo, 30 cm de ancho y 25 cm de altura, donde se colocaron tres ratas en cada una y fueron expuestas a ciclos de luz y oscuridad de 12 horas, con agua (marca Cielo®) *ad libitum* y alimentación balanceada.

Población de estudio y muestra

La población estuvo conformada por 30 ratas albinas machos de variedad Holtzman *Rattus norvegicus* con un peso de 190 ± 7,4 g, adquirida del bioterio del Instituto Nacional de Salud. Se incluyeron ratas sanas, machos, con tres meses de edad. Fueron excluidos dos ratas con comportamiento agresivo y con signos que hayan sido manipulados previamente.

*Inducción a diabetes experimental y descripción del experimento*

Para la inducción de la diabetes experimental, se administró aloxano en dos dosis de 80 mg/kg disuelto en buffer citrato 0,3 M pH 4,5; por vía intraperitoneal, con un espacio de 48 horas entre la primera y segunda dosis. Fueron incluidas las ratas cuyos niveles de glucosa sobrepasaron los 200 mg/dL después de un ayuno de 12 horas ^16^.

Para la fase experimental los animales fueron distribuidos en cinco grupos (n=6), recibiendo el siguiente tratamiento por ocho días, por vía orogástrica: 

Grupo I: Agua

Grupo II: metformina 14 mg/kg p.c

Grupo III: zumo de fruta 1 mL/kg p.c 

Grupo IV: zumo de fruta 4 mL/kg p.c

Grupo V: zumo de fruta 16 mL/kg p.c

El nivel de glucosa en sangre fue evaluado en el primer y octavo día, previo ayuno de 12 horas, haciendo una incisión con bisturí en la punta de la cola, previa limpieza y desinfección, desechando la primera gota de sangre, la medición de glucosa se realizó por duplicado, en un equipo de determinación rápida (ACCU-CHEK Instant).

Una vez terminado el proceso experimental los animales fueron eutanizadas con pentobarbital sódico (50 mg/kg vía intraperitoneal) ^17^, luego se realizó laparotomía para extraer el páncreas.

*Análisis Histológico*

El tejido pancreático fue conservado en solución de formol al 10% tamponado (buffer fosfato 0,05 mol/L a pH 7,4) para su posterior estudio histopatológico. Las muestras obtenidas fueron parafinadas, y se empleó la tinción hematoxilina-eosina a fin de caracterizar las alteraciones microscópicas, empleándose la potencia de 40X, las muestras fueron evaluadas por un profesional médico-patólogo, dicha evaluación de daño se expresó en una escala ordinal: leve (+), moderado (++) y severo (+++).

### Análisis estadístico

Los datos fueron ordenados y analizados en el programa SPSS versión 27. Se evaluó la normalidad de las variables, según la prueba de Shapiro Wilk, donde se obtuvo un valor de p>0,05, por lo que se usó estadística paramétrica. Se calculó la media y desviación estándar, como medida de tendencia central y de dispersión. Se empleó la prueba de análisis de varianza (ANOVA), y el estadístico de Levene, para posteriormente realizar las comparaciones mediante la prueba de Tukey.

### Aspectos éticos

El presente estudio consideró los aspectos contemplados por la Ley N.° 30407 «Ley de protección y Bienestar Animal», conforme al artículo 19 del Capítulo V «Tenencia, protección y manejo de animales», que garantizan la mayor protección contra el dolor físico, basadas en las buenas prácticas de manejo, bioseguridad y bioética de acuerdo con la especie animal experimentado ^18^. Además, se cumplieron con dos de los tres principios de la experimentación con los animales, propuesta por Russell y Burch, el cuales son Reducir y Refinar. El presente estudio cuenta con la aprobación del Comité de Ética en Investigación de la Facultad de Medicina de la Universidad Nacional Mayor de San Marcos (código N.° 0053-2022).

## RESULTADOS

### Capacidad antioxidante *in vitro*

La capacidad antioxidante por el método de DPPH muestra que el extracto presenta un IC_50_ de 0,77 mg/mL, mientras que el IC_50_ para el Trolox fue de 5,86 µg/mL, dando un TEAC-DPPH de 7,61 µg/mg de extracto (Figura 1).

**Figura 1:** Curvas de reduccion el 2,2-difenil-1-picrilhidracilo. (A) Extracto hidroetanolico. (B) Trolox.

Por el método de FRAP se observó que el extracto a 2,93 mg/mL y el Trolox a 66,5 µg/mL expresaron una actividad antioxidante equivalente a 328,7 µM de Fe^2+^, siendo el TEAC-FRAP de 22,31 µg/mg de extracto (Figura 2).

**Figura 2:** Curva de reduccion del FRAP. (A) Extracto hidroetanolico. (B) Trolox.

Efecto hipoglicemiante

Previo al periodo de inducción con aloxano las ratas mostraron un nivel de glucosa en ayunas de 64,8 ± 10,4 mg/dL (Shapiro Willk p>0,05).

En los grupos II al V muestra una disminución de los niveles de glicemia al día 8 respecto al primer día de tratamiento. Se observó que el último día del tratamiento los grupos que recibieron la metformina y el zumo a diferentes dosis mostraron un menor nivel de glucosa respecto al grupo I, mostrando menor nivel los grupos IV y V (Tabla 1).

**Tabla 1:** Niveles de glucosa según grupo de tratamiento en ratas inducidas a cuadro diabético por aloxano.

Grupos	Tratamiento	Nivel de glucosa (mg/dL)	
		Día 1*	Día 8**
		Media ± DE	Media ± DE
Grupo I	Agua 2 mL	496 ± 92,1	452 ± 67,8
Grupo II	Metformina 14 mg/kg	487 ± 81,0	139 ± 28,3^(a)(c)^
Grupo III	Zumo sanky 1 mL/kg	431 ± 118	286 ±39,7^(a)(d)^
Grupo IV	Zumo sanky 4 mL/kg	327 ± 75,2	114 ±13,0^(a)(b)(c)^
Grupo V	Zumo sanky 16 mL/kg	393 ± 126	104 ±5,73^(a)(b)(c)^

DE: desviación estándar.

*Prueba de ANOVA=p>0,05, **prueba de ANOVA=p<0,05.

Prueba de Tukey y ANOVA:

(^a^) p<0,01; comparado con el grupo I

(^b^) p<0,01; comparado con el grupo III

(^c^) p<0,01; comparado con el día 1

(^d^) p<0,05; comparado con el día 1

### Resultados del estudio histopatológico

A nivel histológico del tejido pancreático de las ratas inducidas a diabetes experimental con aloxano, identificó la presencia de:

*Grupo I*

Se observó hipocelularidad y atrofia severa a nivel de los islotes de Langerhans (+++), presentó también necrosis moderada multifocal del islote (++), mientras que a nivel acinar se observó necrosis leve (+) con presencia de congestión vascular (Figura 3).

**Figura 3:** Microfotografias del tejido pancreaticos en los grupos de tratamientos.

*Grupo II*

Se observó hipocelularidad y atrofia severa a nivel de los Islotes de Langerhans (+++), así como necrosis moderada multifocal del islote de Langerhans (++), también se observó que los acinos pancreáticos presentaron necrosis difusa moderada (++) (Figura 3).

*Grupo III*

Se observó hipertrofia moderada a nivel del islote (++) y a diferencia de los grupos anteriores, este presentó hipercelularidad leve a nivel de los islotes de Langerhans (+), así como también necrosis moderada multifocal del islote (++), y a nivel acinar se observó la presencia de necrosis difusa moderada (++) acompañado de congestión vascular (Figura 3).

*Grupo IV*

Este grupo se caracterizó por la presencia de hipertrofia e hipercelularidad leve a nivel de los islotes de Langerhans (+), a nivel multifocal se observó necrosis leve (+) y a nivel acinar necrosis difusa moderada (++), se observó también congestión vascular (Figura 3).

*Grupo V*

En este grupo el corte histológico evidenció hipercelularidad leve (+) e hipertrofia leve en el islote de Langerhans (+), a nivel multifocal se evidenció necrosis leve (+), así como a nivel acinar que se observó necrosis leve (+), acompañado de congestión vascular (Figura 3).

## DISCUSIÓN

En el presente estudio se observó la capacidad antioxidante del extracto de pulpa de sanky (*Corryocactus brevistylus*), mediante el método de DPPH, donde el extracto presentó un IC_50_ de 0,77 mg/mL, mientras que el IC_50_ para el trolox fue de 5,86 µg/mL, dando un TEAC-DPPH de 7,61 µg/mg de extracto. Siguiendo el método de FRAP se observó que el extracto a 2,93 mg/mL, presenta una actividad antioxidante equivalente a 328,7 µM de Fe^2+^, siendo el TEAC-FRAP de 22,31 µg/mg de extracto. Se evaluó también el efecto hipoglicemiante del zumo del *Corryocactus brevistylus*, donde se observó que en las dosis de 4 y 16 mL/kg p.c (grupo IV y V, respectivamente) presentaban efecto hipoglucemiante en ratas macho albinas cepa Holtzman con diabetes *mellitus* experimental, respecto al primer día de tratamiento en un período de ocho días consecutivos, observándose que conforme pasa el tiempo el efecto hipoglicemiante del sanky es mayor en ambos grupos, mientras que la dosis de 1 mL/kg p.c del grupo III, no fue suficiente para normalizar los niveles de glicemia. De igual manera se observó hipertrofia y necrosis leve en los islotes de Langerhans en las ratas del grupo IV y V comparado con el grupo control que no recibió tratamiento de sanky y se pudo encontrar hipocelularidad y atrofia severa a nivel de los Islotes de Langerhans.

Este efecto protector encontrado en los grupos experimentales con sanky podría deberse a su gran capacidad antioxidante *in vitro* encontrado en el extracto seco, donde se pudo observar mediante el método DPPH que presentó un IC_50_ de 0,77 mg/mL, el cual es equivalente a TEAC-DPPH de 7,61 µg/mg de extracto, este resultado podría estar relacionado a la presencia de metabolitos presentes en la fruta, tal y como lo detalla Obregón *et al.* donde encontraron que el sanky presentaba 0,57 de vitamina C mg/g de fruta, además de la presencia de polifenoles, estos compuestos han sido estudiados por su capacidad de neutralizar los radicales libres ^8^. Del mismo modo, Matos *et al*. mediante el mismo método (DPPH) encontraron que a mayor contenido de fenoles es mayor la capacidad antioxidante, observando que la actividad antioxidante es dependiente de la concentración de compuestos fenólicos en el extracto ^19^.

Nolazco y Guevara encontraron que la pulpa de sanky presenta una equivalencia de Trolox de 474,8 µg/g de muestra, presentando buena capacidad antioxidante, debido al contenido de vitamina C encontrado 57,1 mg/100 g de muestra, los autores indican que el sanky presenta mayores contenidos de ácido ascórbico respecto a otras frutas cítricas de mayor consumo ^20^.

En nuestro estudio se evaluó el efecto que produce el zumo de sanky en la glicemia de ratas inducidas con aloxano y el efecto que causa en la morfología del páncreas, donde encontramos que los niveles de glicemia obtenidos después de haber administrado aloxano por vía intraperitoneal 80 mg/kg p.c, fueron acordes a la metodología propuesta, logrando una glucosa mayor a 200 mg/dL, también se observó que el grupo I (control), mantuvo valores altos desde el inicio de la inducción hasta finalizar el tratamiento, estos valores encontrados guardan relación con los efectos del aloxano, ya que es considerado uno de los agentes diabetogénicos ampliamente utilizado para producir diabetes experimental en animales, debido a su mecanismo de acción sobre las células β del páncreas, aumentando los niveles de glucosa en plasma como consecuencia a su administración ^21^.

Los resultados observados en el grupo I (control) guardan relación a lo descrito por Vilches *et al*., donde al administrar aloxano a una dosis única de 100 mg/ kg se logró inducir a hiperglicemia con valores por encima de 200 mg/dL, manteniendo estos valores los siete días que duró el tratamiento ^22^, mientras que Sosa *et al*. realizaron esta inducción a una dosis de 150 mg/kg de aloxano para lograr diabetes experimental con glucosa mayor a 250 mg/dL, encontrando diferencias significativas a los 30 min de la ingesta del grupo control y los grupos con tratamientos, pero la variación de la glucosa fue disminuyendo a medida que fue transcurriendo el tiempo, de manera que a los 120 min posprandiales ya no se encontraron diferencias significativas en el porcentaje de variación entre las dosis, lo que denota que los animales experimentales habían regresado a su estado basal ^23^.

En un estudio realizado con ratas diabéticas inducidas con aloxano y tratadas con insulina se pudo observar atrofia severa a nivel de los islotes pancreáticos, adicionalmente, en este mismo grupo se observó un aumento significativo en el número de células pancreáticas (hipercelularidad) ^24^, lo cual difiere de nuestra investigación, donde se observó una disminución de las células pancreáticas (hipocelularidad) en el grupo control y el grupo con tratamiento de metformina. En nuestro estudio a nivel histológico se observó en el grupo I la presencia de atrofia severa a nivel de los Islotes de Langerhans en el grupo I. El daño descrito en el grupo I también fue observado en el grupo de ratas que recibió metformina, fármaco utilizado como hipoglicemiante, también presentó atrofia severa a nivel de los Islotes.

Las ratas tratadas con zumo de sanky a dosis altas de 4 mL/kg y 16 mL/kg mostraron una mayor protección de las células pancreáticas, y se observó hipertrofia leve a nivel de los Islotes, demostrando que este producto además de disminuir los niveles de glucosa en sangre brindaría una mejor protección a las células del páncreas.

El efecto que presentó el zumo del sanky sobre las células pancreáticas podría deberse al efecto antioxidante que éste presenta, así como el contenido de metabolitos que le dan un valor apreciable desde el punto de vista nutricional y como posible alimento funcional, para atenuar los efectos deletéreos de la generación de radicales libres, mediante su capacidad antioxidante reportada por Balvin en el 2021 ^25^.

El grupo que recibió, además de aloxano, el sanky en dosis mínima de 1 mL/kg, mostró signos de necrosis moderada, lo que indicaría una mínima protección a diferencia de las ratas que recibieron la dosis más alta, que se observó una leve atrofia en las células pancreáticas.

Debido a que el presente estudio fue realizado en animales de experimentación, los resultados no podrían extrapolarse al ser humano, pese a encontrar beneficios en dicha fruta, ya que aún es base de futuras investigaciones. También es importante identificar los compuestos bioactivos presentes en el sanky, así como se pudo conocer el efecto antioxidante que presenta, es necesario determinar el metabolito responsable de los efectos beneficiosos. Cabe mencionar que los antioxidantes se encuentran presentes en muchos alimentos, y de acuerdo con la literatura, los antioxidantes pueden neutralizar el exceso de radicales libres durante la actividad oxidativa, propia del organismo, actuando como primera línea de defensa de los seres vivos, sin embargo, un desbalance entre antioxidantes endógenos y radicales libres (estrés oxidativo) se asocia con diferentes enfermedades ^26^.

Por todo lo mencionado con anterioridad podemos destacar al sanky como una fruta con altas propiedades benéficas para el organismo, siendo base para la elaboración de productos derivados que beneficiarían a la población en general, así como derivados nutraceúticos, cremas o suplementos que conserven sus propiedades antioxidantes, y que sea de fácil acceso para la población.

Podemos concluir, de todo lo observado en el presente estudio de investigación, que el sanky presenta un efecto antioxidante *in vitro*, efecto hipoglicemiante y protector del tejido pancreático en dosis de 4 y 16 mL/kg a nivel pancreático en ratas inducidas a diabetes con aloxano.
